# Response of bean cultures’ water use efficiency against climate warming in semiarid regions of China

**DOI:** 10.1016/j.agwat.2016.05.010

**Published:** 2016-07-31

**Authors:** Xiao Guoju, Zhang Fengju, Huang Juying, Luo Chengke, Wang Jing, Ma Fei, Yao Yubi, Wang Runyuan, Qiu Zhengji

**Affiliations:** a New Technology Application R&D Center of Ningxia University, Yinchuan, Ningxia 750021, China; b Institute of Arid Meteorology, CMA, Key Laboratory of Arid Climatic Change and Disaster Reduction of Gansu Province, Lanzhou, Gansu 730020, China; c Ningxia Liupanshan Flower Research Institute, Longde, Ningxia 756300, China

**Keywords:** Climate change, Broad bean, Yield, Water use efficiency, Semiarid regions

## Abstract

Farm crop growing and high efficiency water resource utilizing are directly influenced by global warming, and a new challenge will be given to food and water resource security. A simulation experiment by farm warming with infrared ray radiator was carried out, and the result showed photosynthesis of broad bean was significantly faster than transpiration during the seedling stage, ramifying stage, budding stage, blooming stage and podding stage when the temperate was increased by 0.5–1.5 °C. But broad bean transpiration was faster than photosynthesis during the budding stage, blooming stage and podding stage when the temperature was increased by 1.5 °C above. The number of grain per hill and hundred-grain weight were significantly increased when the temperature was increased by 0.5–1.0 °C. But they significantly dropped and finally the yield decreased when the temperature was increased by 1.0 °C above. The broad bean yield decreased by 39.2–88.4% when the temperature was increased by 1.5–2.0 °C. The broad bean water use efficiency increased and then decreased with temperature rising. The water use efficiency increased when the temperature was increased by 1.0 °C below, and it quickly decreased when the temperature was increased by 1.0 °C above. In all, global warming in the future will significantly influence the growth, yield and water use efficiency of bean cultures in China’s semiarid regions.

## Highlights

• Global warming will significantly influence the yield and water use efficiency of bean cultures.
• Transpiration was faster than photosynthesis when the temperature was increased by 1.5 °C above.
• The yield decreased by 39.2–88.4% when the temperature was increased by 1.5–2.0 °C.
• The water use efficiency quickly decreased when the temperature was increased by 1.0 °C above.

In the recent half a century, the climate of semiarid regions in Northwest China has experienced changes of high temperature, arid, warm winter, etc, thus the air temperature significantly increased, the rainfall generally decreased, and warming with drought became more and more distinct. Especially in the recent 30 years, drought and water deficiency were enlarged and aggravated, and drought was so severe and long lasting in some regions that it had never happened before. Climate change affects agriculture and water resource more and more significantly and seriously (Du et al., 2012). In the coming 50 years, farm crop growing and high efficiency water resource utilizing are directly influenced by global warming, and a new challenge will be given to food and water resource security (Ding et al., 2009). The increasingly severe drought has brought the arid agriculture a critical problem in rainwater resource availability research for utilizing the limited rainwater resource effectively (Zhang et al., 2005, Singh et al., 2014).

Improving the water use efficiency is a key factor for continuously increasing the crop yield in arid regions in Northwest China. A lot of research was made by Chinese and foreign scholars for the effect of climate change on crop water availability. Ogaya and Peuelas (2003) found crops in arid and semiarid regions maintained a higher water availability to reduce the effect of water deficiency and enhance the competitiveness for moisture in drought. Tenhunen et al. (2002) researched and found that climate warming accelerated crop transpiration and soil moisture evaporation, and influenced the crop water use efficiency in arid and semiarid regions. Water use efficiency in the crop ecological system drops with the decrease of soil moisture, and it means the crop photosynthesis changes down by some other factors besides the stomatal factor. Zhao et al. (2007) researched and found that the net photosynthesis and stomatal conductance of leaves of spring wheat during the grain filling stage and milk stage in semiarid regions dropped and the transpiration increased with air temperature rising, the photosynthesis and dry substance accumulation were inhibited by climate warming, as a result, the crop water use efficiency was degraded.

The soil fertility in China’s arid and semiarid regions is seriously poor. Planting bean cultures which can fertilize the soil by the nitrogen fixation of rhizobia is an effective way for promoting the crop growth and improving the grain yield in China’s semiarid regions. Continuous cropping or interplanting with bean cultures and gramineous cultures is a key planting mode in China’s semiarid regions as it obviously increases the yield and improves the soil nitrogen availability. Broad bean is a great annual bean culture commonly planted in China’s semiarid regions as it has a series of favorable biological properties widely adapted to the environment against coldness, droughts, waterlogging, plant diseases and insect pests, and is rich in nutrition. This paper uses the large farm infrared radiator warming method to realize a research of warming effect on broad bean water use efficiency and provide a scientific reference for improving the broad bean climatic adaptation and control measures.

## 1 About the base station

**Fig. 1:**
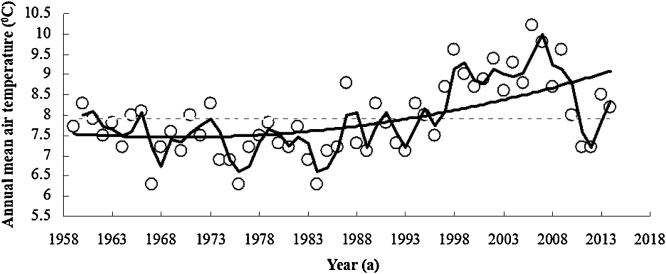
Air temperature change in the semiarid Guyuan area during 1960–2014.

The experiment was made in Guyuan Experimental Station in a typical China’s semiarid region at N35.14″–36.38″ and E105.20″–106.58″. The annual air temperature during 1960–2014 was 6.3 °C–10.2 °C and the multi-year mean air temperature was 7.9 °C. The air temperature distinctly rose in the recent 50 years and especially after 1998 (Fig. 1). The annual rainfall volume during 1964–2014 was 282.1–765.7 mm and the multi-year mean rainfall was 450.0 mm. The rainfall volume in the recent 50 years was distinctly decreasing. Wheat, broad bean and corns, etc are main crops matured once per year, and the region is a typical semiarid rainfall farming area.

## 2 Experimental design and method

### 2.1 Simulated warming test

The research for warming effect on broad bean water use efficiency was done by field infrared radiator warming methods (Xiao et al., 2013). In December 2009 on the United Nations Climate Change Conference held in Copenhagen— the capital city of Denmark, it fixed target that the global warming amplitude in the coming 50 years will be controlled at 2.0–2.4 °C (Zheng, 2010). Therefore, the designed warming stages were 0 °C, 0.5 °C, 1.0 °C, 1.5 °C and 2.0 °C. Each plot in the experimental farm was 8 m^2^ (2m × 4m) and plots were spaced for 3.0 m. Each plot was equipped with 2 infrared radiator warming tubes, and the support height was adjusted so that the warming pipe was spaced from the crop canopy height for 1.2 m. The warming pipe power was fixed according to the warming requirement and local air temperature. The infrared radiator warming pipe powers used in the experiment were 250 W, 500 W, 750 W, 1000 W, 1250 W and 1500 W respectively. Broad beans were warmed continuously at day and night during the whole growing period (from seedling emergence to harvest). The experimental farm soil was loessal soil with 8.5 g of organic matter, 0.41 g of total nitrogen, 0.66 g of total phosphorus and 19.5 g of total potassium per kg. The experimental farm was fenced at four sides to prevent animals.

### 2.2 Water use efficiency calculation

Farm water consumption was calculated based on the soil moisture data collected in the broad bean seedling stage, ramifying stage, budding stage, blooming stage and podding stage.

ET_1–2_ = Σg_i_ H_i_(θ_i1_–θ_i2_) + P_0_ + M + K, (i = 1,2,…,n)

In the equation above, ET_1-2_ is the stage water consumption, i is the soil layer number, n is the total number of layers; γi is the soil dry bulk density of soil layer i, H_i_ is the thickness of soil layer i, θ_i1_ and θ_i2_ are respectively the stage beginning moisture content and stage end moisture content of soil layer i, counted by the percentage of the dry soil weight, p_0_ is the effective rainfall volume, M is the water irrigated in a time section; and K is the moisture content compensated by the underground water in a time section. When the underground water is deeper than 2.5 m, K can be neglected; crops in the researched region are not irrigated, the underground water is deeper than 5 m, so M and K can be neglected. Water use efficiency was calculated as per the equation below.

WUE = Y/ETα

In the equation, WUE is the water use efficiency (kg ha^−1^ mm^−1^), *Y* is the yield (kg ha^−1^), ETα is the actual moisture consumption (mm) during the crop growing stage, i.e. sum of moisture consumption in all stages.

### 2.3 Data processing method and monitoring

Crops were harvested manually and the yield was measured and recorded actually. Soil moisture content was measured by aluminum box drying method. Soil was sample by a soil drill, each 20 cm was a layer, and the soil sampling depth was 0–100 cm. Each soil sample was put into an aluminum box at the first time and dried to a constant weight at 110 °C before the soil moisture content was calculated.

During the broad bean whole growing and warming period, each plot was equipped with an automatic temperature sensor to detect the air temperature at 10 cm, 20 cm and 30 cm to the ground or canopy layer once per 20 min, and results were automatically output and saved in the recorder (Campbell AR5, error ±0.1 °C)

Crop yield, soil moisture content, rainfall volume, air temperature and other agricultural and meteorological data were processed and mapped by Microsoft Excel 2003.

## 3 Result and analysis

### 3.1 Warming effect on photosynthesis

**Fig. 2:**
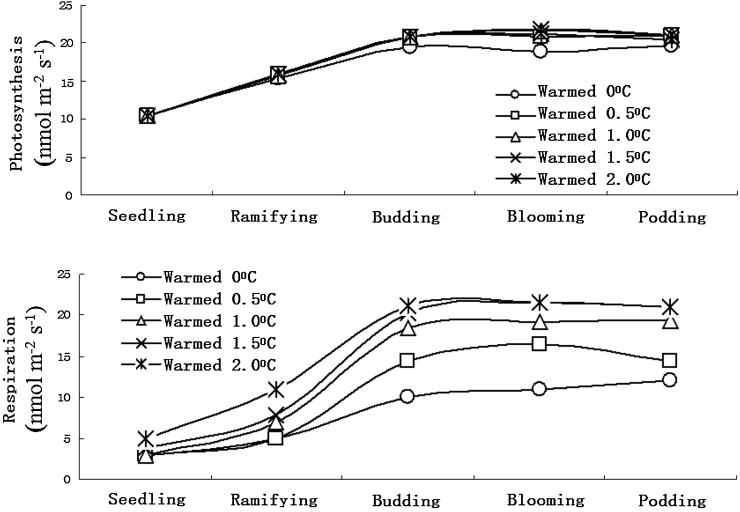
Warming effect on broad bean photosynthesis and transpiration.

Broad beans have different features and different requirements for ambient conditions in different growing stages. In the budding stage, dry matter forms and accumulates a lot, and it is also the nutriment growing stage and reproductive growing stage. Temperature influences a lot to the time of broad bean ramifying and budding, as excessively tall plants in the budding stage may bring too much shadow which may cause excessive falling off of pods and lodging of crops. Excessively short plants are not good for rich yield. Broad bean blooming and podding are simultaneous, the blooming and podding stages are the most important growing stages when organs compete for the assimilation product the most severely. This research shows the photosynthesis and transpiration of broad bean in China’s semiarid regions significantly accelerate in the seedling stage, ramifying stage, budding stage, blooming stage and podding stage (Fig. 2). With temperature rising, i.e. warmed to 0 °C, 0.5 °C, 1.0 °C, 1.5 °C and 2.0 °C, the broad bean photosynthesis doesn't change too much, but the transpiration changes obviously.

**Fig. 3:**
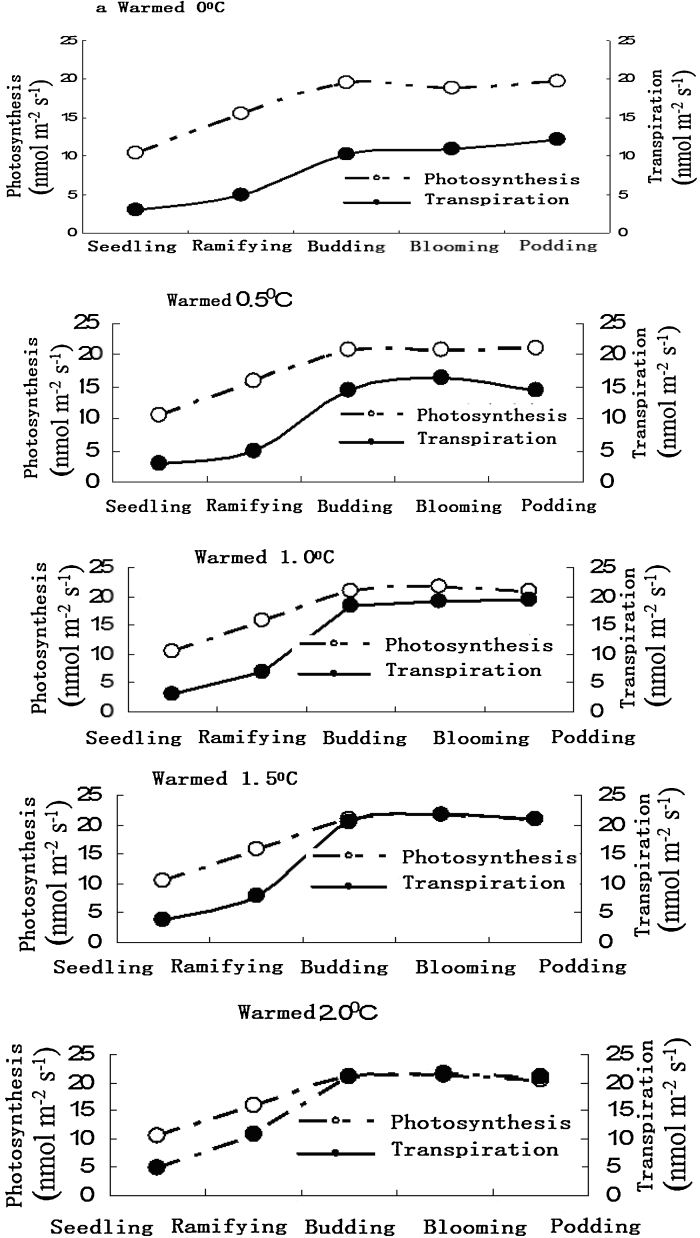
Change of broad bean photosynthesis and transpiration in different warming conditions.

Broad bean photosynthesis and transpiration changes in different warming conditions, when warmed by 0.5–1.5 °C, photosynthesis is distinctly faster than transpiration. When warmed by 1.5 °C above, broad bean photosynthesis at the seedling stage and ramifying stage is distinctly faster than transpiration, but transpiration is faster than photosynthesis in the budding stage, blooming stage and podding stage (Fig. 3).

### 3.2 Warming effect on yield

Drought in broad bean flowering and blooming stage mainly affects the number of pods and number of heavy kernels. Drought in the podding stage and full podding stage decreases the hundred-grain weight. Drought in the blooming and podding stage sharply decreases the yield. Broad bean yield is determined by the number of harvested plants, number of kernels per plant, and the hundred-grain weight. Table 1 shows that warming distinctly affects the number of kernels per plant and hundred-grain weight. Further warming distinctly increases the broad bean number of kernels per plant and hundred-grain weight. But when warmed to 1.5 °C above, number of kernels per plant and hundred-grain weight distinctly drop and caused to a yield decrease. Warming for 0.5–1.0 °C distinctly increases the broad bean yield by 12.9%–16.1%. But the yield decreases by 39.2–88.4% when the temperature was increased by 1.5–2.0 °C.

### 3.3 Warming affect on water use efficiency

**Fig. 4:**
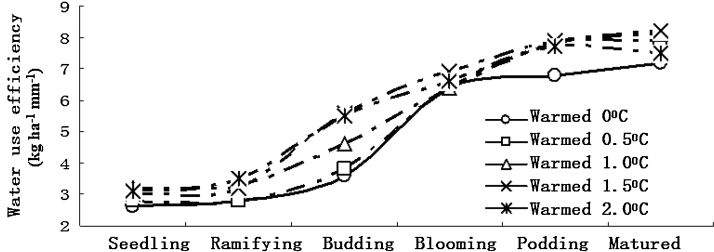
Warming effect on broad bean water availability at different growing stages.

Fig. 4 shows that the water use efficiency distinctly increased in the broad bean seedling stage, ramifying stage, budding stage, blooming stage and podding stage (Fig. 2). With warming, i.e. warmed to 0.5 °C, 1.0 °C, 1.5 °C and 2.0 °C, the broad bean water use efficiencies are distinctly higher than those in the unwarmed stage.

**Fig. 5:**
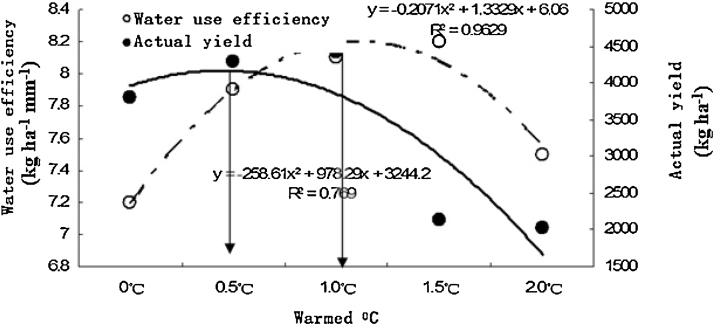
Correlation of warming to broad bean yield and water use efficiency.

Fig. 5 shows that with warming, i.e. warmed to 0.5 °C, 1.0 °C, 1.5 °C and 2.0 °C, the broad bean yield and water use efficiency increases before decreasing. Broad bean yield increases when warmed to 0.5 °C below and decreases when warmed to 0.5 °C above. The water use efficiency increased when the temperature was increased by 1.0 °C below, and it quickly decreased when the temperature was increased by 1.0 °C above. Climate warming will significantly affect broad bean growth and yield.

## 4 Discussion

Climate warming significantly affects the water use efficiency via modifying the plant productivity and evaporation. Warming affects the plant evaporation via modifying the stomatal conductance. Below a certain threshold, warming increases the leaf stomatal conductance, net photosynthesis increases faster than transpiration, and thus the water use efficiency is improved; above a certain threshold, warming increases evaporation and further decreases the water use efficiency. Yepez et al. (2007) made a general observation on the semiarid mesquites, and they found warming affected evaporation significantly modified the water use efficiency. Gao et al., 2007a, Gao et al., 2007b researched and found that leaf temperature rising of sorghum sudanense in the arid region improved the net photosynthesis and transpiration, and caused a significant negative correlation between single leaf water use efficiency and leaf temperature. Tenhunen et al. (2002) researched and found that climate warming accelerated crop transpiration and soil moisture evaporation, and influenced the crop water use efficiency in semiarid regions. Water use efficiency in the crop ecological system drops with the decrease of soil moisture, and it means, under the extremely arid condition, the crop photosynthesis changes down by some other factors besides the air pore factor. Zhao et al. (2007) researched and found that climate warming inhibited photosynthesis and dry matter accumulation and further influenced the water use efficiency of spring wheat in the northwest semiarid region. Wang et al. (2010) researched and found that in the northwest semiarid region, climate warming decreased the water use efficiency of main crops corn and spring wheat, the corn and spring wheat water use efficiencies decreased with an index or parabola curve with the increase of moisture supply in the growing period, and warming is negative to crop water use efficiency.

Warming is good for crop photosynthesis and can improve the crop water use efficiency. Loader et al. (1995) verified that warming increased plant photosynthesis and further promoted the plant water use efficiency. Yao et al. (2011) researched and found that winter and spring air temperature in the semi-humid arid region of Loess Plateau significantly increased, winter wheat over-winter death rate distinctly dropped, and the water use efficiency rose. Xiao et al. (2013) researched and found that water use efficiencies of spring wheat, potato and corn in the northwest semiarid region increased with air warming in the past 50 years. However, excessively warming affected crop photosynthesis, increased transpiration and soil moisture evaporation, and decreased the crop water use efficiency (Wang et al., 2010). Jiang and Dong (2000) researched and found that aggravating drought gradually increased the plant water use efficiency but decreased it above a certain threshold. Below a certain threshold, warming increases the leaf stomatal conductance, net photosynthesis increases faster than transpiration, and thus the water use efficiency is improved (Shen et al., 2002); above a certain threshold, warming increases evaporation and further decreases the water use efficiency (Wang, 2009, Yin et al., 2009).

Warming affects crop photosynthesis, transpiration and soil moisture evaporation, and further affects crop water use efficiency. A higher temperature brings stronger crop transpiration and soil moisture evaporation, and may decrease the crop water use efficiency (Zhao and Yu, 2008). Loader et al. (1995) verified that warming improved the photosynthesis and further improved the water use efficiency of stalkless flowered oak seedlings. Gao et al., 2007a, Gao et al., 2007b researched and found that leaf temperature rising of sorghum sudanense in the arid region improved the net photosynthesis and transpiration, and caused a significant negative correlation between single leaf water use efficiency and leaf temperature. The temperature effect on plant water use efficiency is more or less complicated. Warming affects the plant transpiration via modifying the stomatal conductance. Below a certain threshold, warming increases the leaf stomatal conductance, net photosynthesis increases faster than transpiration, and thus the water use efficiency is improved; above a certain threshold, warming increases evaporation and further decreases the water use efficiency (Schleser, 1990). The research result of this paper shows that the water use efficiency of broad bean in China’s semiarid regions rises and then drops with air warming. When warmed by 0.5–1.5 °C, the broad bean water use efficiency distinctly increases. But when warmed by 1.5 °C above, it distinctly decreases.

When researching the plant water use efficiency, plant respiration features can be more favorably explained if the effects of moisture conditions, temperature and other factors are put into consideration, and thus the plant water use efficiency can be recognized more exactly (Tenhunen et al., 2002). In view of plant physiology, the change of plant water use efficiency in drought is caused by stomatal restrictive factors and non-stomatal restrictive factors. Stomatal restriction was realized by the regulation of leaf air bores protecting the cell movement, when the plant is under a light or medium moisture threat, air bores will be more sensitive to drought, the net photosynthesis is non-linear to the stomatal conductance which decreases before the decrease of net photosynthesis, and thus the transpiration is decreased, the water use efficiency is improved (Picotte et al., 2007, Richards et al., 2014). Effective moisture in arid and semiarid regions is the most important factor for controlling the plant function, and the decrease of effective moisture will aggravate the plant physiological threat and weakness. Ogaya and Peuelas (2003) researched and found crops maintained high water availability in drought to reduce the effect of water deficiency and enhance the competitiveness for moisture in drought.

## 5 Conclusion

Aridification in semiarid regions of Northwest China aggravated obviously in the past 50 years. In the coming 50 years with global warming, crop photosynthesis will be directly affected, crop transpiration and soil moisture evaporation will be highly increased, and thus crop growing will be inhibited, yield and water resource will be degraded, and a new challenge will be thrown to food and water resource security.

The simulated experiment of farm warming by infrared radiator shows that broad bean photosynthesis and transpiration changes differently in different warming conditions. When warmed by 0.5–1.5 °C, the broad bean photosynthesis was faster than transpiration. But when warmed by 1.5 °C above, the broad bean transpiration in the budding stage, blooming stage and podding stage was faster than photosynthesis, and warming distinctly affected photosynthesis.

Broad bean yield is determined by the number of harvested plants, number of kernels per plant, and the hundred-grain weight. Warming distinctly affects the number of kernels per plant and hundred-grain weight. Further warming distinctly increases the broad bean number of kernels per plant and hundred-grain weight, but when warmed to 1.5 °C above, the number and weight distinctly drop and caused to a yield decrease. The yield decreases by 39.2–88.4% when the temperature was increased by 1.5–2.0 °C.

The broad bean yield and water use efficiency increased and then decreased with temperature rising. Broad bean yield increased when warmed to 0.5 °C below and decreased when warmed to 0.5 °C above. The water use efficiency increased when the temperature was increased by 1.0 °C below and it decreased when the temperature was increased by 1.0 °C above. In all, growth and yield of bean cultures in the semiarid regions of Northwest China will be significantly affected by warming.

## Figures and Tables

**Table 1:** Warming effect on broad bean yield and yield composition.

Warmed (°C)	Number of plants harvested (10,000 plants ha^−1^)	Number of pods per plant	Number of kernels per pod	Number of kernels per plant	Hundred-grain weight (g)	Yield (t ha^−1^)	Yield increased or decreased (%)
0	6.18a	5.6a	7.3a	40.8a	163.0a	3804.0a	/
0.5	6.19a	5.8a	8.5a	49.3b	160.8a	4297.5b	+12.9
1.0	6.19a	6.2b	8.4a	52.1c	165.3a	4417.5b	+16.1
1.5	6.19a	4.3b	6.3a	27.1b	162.3a	2314.0b	−39.2
2.0	6.19a	4.1b	5.8b	23.8b	152.6b	2019.0b	−88.4

*Note*: Letters in each column means significant differences under 5%, and “a” is presented with a significant difference in respect of “b”.
